# Characterization of the resistance to Vip3Aa in *Helicoverpa armigera* from Australia and the role of midgut processing and receptor binding

**DOI:** 10.1038/srep24311

**Published:** 2016-04-20

**Authors:** Maissa Chakroun, Núria Banyuls, Tom Walsh, Sharon Downes, Bill James, Juan Ferré

**Affiliations:** 1 ERI of Biotechnology and Biomedicine (BIOTECMED), Universitat de València, 46100 Burjassot, Spain; 2 CSIRO, Black Mountain Laboratories, Canberra, ACT 2601, Australia; 3 CSIRO, Myall Vale Laboratories, Kamilaroi Highway, Narrabri, NSW 2390, Australia

## Abstract

Crops expressing genes from *Bacillus thuringiensis* (Bt crops) are among the most successful technologies developed for the control of pests but the evolution of resistance to them remains a challenge. Insect resistant cotton and maize expressing the Bt Vip3Aa protein were recently commercialized, though not yet in Australia. We found that, although relatively high, the frequency of alleles for resistance to Vip3Aa in field populations of *H. armigera* in Australia did not increase over the past four seasons until 2014/15. Three new isofemale lines were determined to be allelic with previously isolated lines, suggesting that they belong to one common gene and this mechanism is relatively frequent. Vip3Aa-resistance does not confer cross-resistance to Cry1Ac or Cry2Ab. Vip3Aa was labeled with ^125^I and used to show specific binding to *H. armigera* brush-border membrane vesicles (BBMV). Binding was of high affinity (*K*_*d*_ = 25 and 19 nM for susceptible and resistant insects, respectively) and the concentration of binding sites was high (*R*_*t*_ = 140 pmol/mg for both). Despite the narrow-spectrum resistance, binding of ^125^I-labeled Vip3Aa to BBMV of resistant and susceptible insects was not significantly different. Proteolytic conversion of Vip3Aa protoxin into the activated toxin rendered the same products, though it was significantly slower in resistant insects.

*Helicoverpa armigera* Hübner (Lepidoptera: Noctuidae) is a polyphagous pest feeding on more than 180 species of plants of which the most economically important are grain sorghum, corn and cotton^1^,^2^. It has one of the widest distributions of any agricultural pest, until recently being confined to Asia, Europe, Africa and Australia. It was considered a quarantine agricultural pest with no successfully established population in the American continent until 2013 when its intrusion to at least twelve Brazilian states caused economic losses up to 4 billion US dollars^2^ and in 2015 was detected in pheromone traps in Florida, USA^3^. *Helicoverpa armigera* is known for its high level, rapid development of resistance to synthetic insecticides^4^,^5^,^6^. For better control of this pest, *Bt* crops expressing insecticidal proteins from *Bacillus thuringiensis* have been commercialized globally. China grows first generation *Bt*-cotton expressing Cry1Ac^7^, while Australia, India and the US introduced dual toxin plants expressing Cry1Ac and Cry2Ab^8^,^9^,^10^. Even before the release of dual toxin cotton in Australia, research showed higher than expected levels of resistance alleles in *H. armigera* and *Helicoverpa punctigera* to one of the Bt proteins (Cry 2Ab)^11^,^12^, and resistance risk remains a critical concern. In addition, various field populations of major lepidopteran pests have now been reported to have developed resistance to Bt crops^10^,^13^,^14^.

The Vip (vegetative insecticidal proteins) proteins discovered in 1996 from *Bacillus cereus* and *B. thuringiensis* are compatible with Cry proteins for insect control because they do not share sequence homology and presumably have different modes of action^15^. Several members of the Vip3A family have high activity against lepidopteran pests^16^. The *vip3Aa* gene has been introduced in plants and was first expressed as a single insecticidal protein in cotton^17^, and later used as a pyramided protein in combination with other *cry* genes, in cotton and in corn, to confer higher protection and delay insect resistance (http://www.epa.gov/oppbppd1/biopesticides/pips/pip_list.htm). However, a significant threat to Bt-based insect control is the potential development of insect resistance that could jeopardize their-long term success. Resistance to Vip3Aa has already been selected in laboratory colonies of at least three heliothine species. Selection for Vip3Aa resistance in *Heliothis virescens* over 13 generations resulted in insects with a resistance ratio of 2040-fold relative to susceptible insects^18^. Of more concern, F_2_ screens detected frequencies of alleles conferring resistance to Vip3Aa as high as 0.03 and 0.01, respectively, in field populations of *H. armigera* and *H. punctigera* from Australia that had not been exposed to plants expressing the toxin^19^.

Vip3A is an intestine specific virulence factor; after being ingested, the proteins are processed by the insect midgut proteases^20^,^21^,^22^,^23^. The major proteolytic products of Vip3Aa are approximately 62 and 20 kDa fragments that are inseparable in size-exclusion chromatography^24^. The processed protein binds to its specific receptors in the midgut epithelial brush border membrane and forms pores^20^,^21^,^24^. Although both Vip3 and Cry proteins need to be activated and bind to membrane receptors to exert their toxic action, the two proteins display different levels of stability and processing rates in the insect midgut^23^ and bind to different specific receptors in the BBMV of the susceptible insects^24^,^25^,^26^. These differences in the mode of action are thought to be responsible for the absence of cross-resistance to Vip3 proteins observed in Cry-resistant insects from all insect species tested^27^,^28^,^29^,^30^,^31^,^32^,^33^,^34^. However, studies on cross-resistance to Cry proteins in Vip3-resistant insects are lacking.

The mechanisms underlying resistance to the Cry proteins have been studied in some field resistant populations and laboratory selected populations. In many cases the gene and mutation responsible has been identified. For Cry1Ac, a number of mutations in resistant individuals have been identified as responsible for phenotypic resistance^35^ and recently genes involved in Cry1Ca resistance in *Spodoptera exigua* and in Cry2Ab resistance in *H. armigera* were also isolated^36^,^37^. In addition, gene expression alterations have also been characterized in some other cases^38^,^39^. Most cases of insect resistance to Cry proteins reported to date belong to one of the sequential steps proposed for their mode of action: impaired proteolysis activation^40^,^41^ or decreased binding to midgut receptors^42^,^43^. However, there are a few cases of resistance to Cry proteins that could not be associated with either impaired activation or decreased binding^44^,^45^,^46^.

The first resistance alleles to Vip3A in field populations of Australian *H. armigera* were isolated in 2009. Pooling F_2_ screen data across 2009/10 and 2010/11 yielded an *r* frequency for *H. armigera* of 0.027 (28 positive lines, 273 tested lines) with a 95% CI between 0.019 and 0.038. Complementation tests involving crosses of the first two isolates (SP85 and SP477) demonstrated that the F_1_ progeny were also resistant to Vip3A, implying that the resistance in both isolates is due to alleles at a common locus. Characterization of these early isolations showed that the resistance to Vip3Aa is recessive and maps to a locus different from that conferring resistance to Cry2Ab^19^,^33^. Herein, in addition to providing up-to-date information on frequencies of resistance alleles in *H. armigera* field populations and further information on allelism among different resistant families, we provide one of the first demonstrations of a lack of cross-resistance to Cry1Ac and Cry2Ab in a Vip3Aa resistant colony. These studies provide important context to a further investigation reported herein which examines the possible role of Vip3Aa processing and binding to midgut receptors as mechanisms of resistance.

## Results

### Characteristics of Vip3Aa resistance

Complementation tests involve crossing a standard resistant colony with the new isolate and then testing the offspring by exposing them to the discriminating dose of the toxin. If the offspring survive the discriminating dose of Vip3Aa, then the resistance in each colony is due to the same mutation or variants (alleles) at the same gene which implies a common mechanism. If a complementation test performed on a new resistant isolate is negative (the offspring of a cross between it and the standard colony fail to survive), it is likely that different genes are involved in conferring resistance.

Previously we reported data for two isolations of *H. armigera* which were allelic with SP85 – here we test an additional four isolations from two seasons of monitoring (2011/2012 and 2012/2013) using F_2_ tests. Three of these Vip3Aa isolations were found to be clearly allelic to the resistant laboratory line SP85 (11–1112, 12–2602, 12–2998). In one line (11–2201) there was substantial mortality from Vip3Aa (~60%) in the offspring from the crosses to SP85 when compared to the control. This could be explained if another gene is involved in conferring the resistance or the tested individuals were heterozygous which would produce approximately 50% mortality. Unfortunately it was not possible to maintain the 11–2201 line to investigate this issue further. However, in all cases the survival rates are greater than would be expected if no resistance allele was present (p = <0.001 χ^2^ = 171432). This result supports the notion of a relatively common mechanism for Vip3Aa resistance in field populations of *H. armigera* in Australia, and justifies using F_1_ screens to estimate SP85-like Vip3Aa resistance frequencies (Table 1). However, it would be prudent to continue to perform some F_2_ screens to track whether resistance involving other potential mutations increases in frequency after the deployment of plants expressingVip3Aa.

Mahon *et al.*^19^ reports frequencies of Vip3Aa resistance alleles for 2009/10 and 2010/11 based on F_2_ screens. Here we report F_2_ screen data from 2011/12 to 2012/13, and F_1_ screen data from 2013/14 and 2014/15. This reflects a shift in the approach used for resistance monitoring. Since the allelism data show one common form of Vip3Aa resistance, in 2013/14 we shifted our focus to the common resistance using the more efficient F_1_ screen (this shift is outlined in more detail in Walsh *et al.*^33^).

F_2_ screens in 2011/12 and 2012/13 estimated an *r* frequency for Vip3Aa in *H. armigera* of 0.025 with a 95% CI between 0.017 and 0.036 (14 positive lines, 284 tested lines) and 0.025 with a 95% CI between 0.016 and 0.037 (12 positive lines, 242 tested lines) respectively. There is no statistically significant difference (Fisher’s Exact test, P = 0.05) between these estimates and those obtained in 2009/10 (0.029, 11 positive lines, 108 tested lines) and 2010/11 (0.028, 17 positive lines, 165 tested lines). Summed across the four years the estimated *r* frequency for Vip3Aa in *H. armigera* based on F_2_ screens is 0.034 with a 95% CI between 0.022 and 0.037 (54 positive lines, 799 tested lines) and has not changed between 2009–2013.

F_1_ screens in 2013/14 and 2014/15 estimated an *r* frequency for Vip3Aa in *H. armigera* of 0.009 with a 95% CI between 0.002 and 0.018 (6 positive lines, 321 tested lines) and 0.016 with a 95% CI between 0.007 and 0.025 (10 positive lines, 313 tested lines) respectively; there is no statistically significant difference between these approximations (Fisher’s Exact test, P < 0.05). Summed across both years the estimated *r* frequency for Vip3Aa in *H. armigera* based on F_1_ screens is 0.013 with a 95% CI between 0.006 and 0.019 (16 positive lines, 634 tested lines).

As part of F_2_ screens performed during the monitoring program from 2009/10 to 2012/13 we examined cross-resistance against Cry1Ac and Cry2Ab by screening isofemale families of *H. armigera* that scored positive for carrying a resistance allele for Vip3Aa. Table 2 summarizes these data and shows that none of the randomly selected 16 families examined showed a greater propensity for survival against Cry1Ac and Cry2Ab toxin than did a Vip3Aa susceptible laboratory colony. The sample is representative of the 54 families of *H. armigera* that scored positive for carrying a resistance allele for Vip3Aa. We therefore conclude that larvae resistant to Vip3Aa are not cross-resistant to Cry1Ac of Cry2Ab.

### Vip3Aa processing with midgut juice of susceptible and resistant *H. armigera*

**Figure 1:**
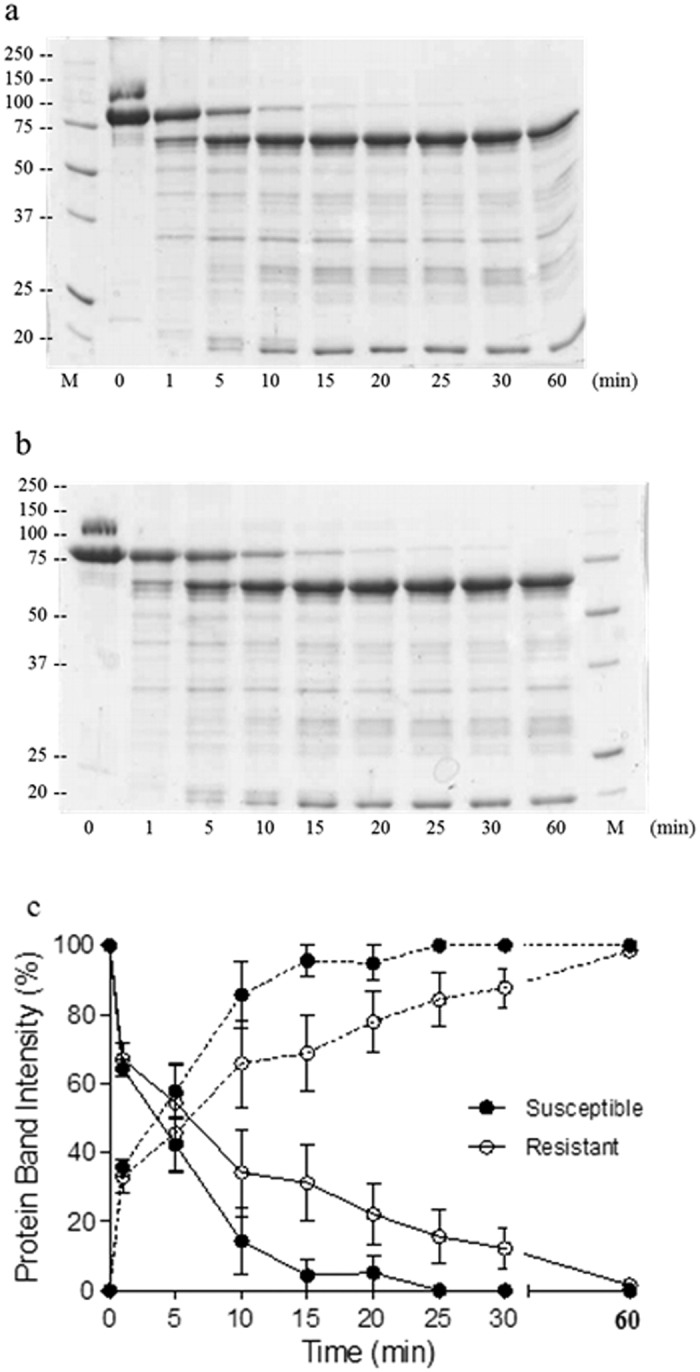
Kinetics of the proteolytic processing of Vip3Aa incubated with midgut juice from *H. armigera* larvae. Incubations were performed at 30 °C and 0.1% of midgut juice total protein referred to Vip3Aa protein. Samples from susceptible (**a**) and resistant (**b**) insects were subjected to SDS-PAGE at different time intervals and the bands of protoxin (89 kDa, solid lines) and activated protein (62 kDa, broken lines) were quantified by densitometry (**c**). GR (susceptible) (●) and SP85 (resistant) (○) colonies. Data points represent the mean of three replicates with the standard error indicated by error bars.

Since Vip3Aa is found in the protoxin form in cotton leaves^47^, we searched for differences in its conversion to the activate form between the susceptible and resistant insects. When midgut juice of GR and SP85 was incubated with Vip3Aa protoxin, many proteolytic products were obtained but no difference in the band profile between the two colonies was observed (Fig. 1a,b). The major proteolysis products were the 62 and the 20 kDa fragments in both cases. The kinetic analysis of the 89 kDa activation and the 62 kDa fragment formation showed a difference in the processing rate between the susceptible and the resistant *H. armigera* colonies (Fig. 1c). The processing of the 89 kDa protoxin was faster in the susceptible colony. After 15 min the protoxin completely disappeared with the midgut juice from the susceptible insects, however, with SP85 there was 31% residual protoxin which was completely activated after 60 min incubation.

### ^125^I-Vip3Aa binding to the BBMV of susceptible and resistant *H. armigera*

**Figure 2:**
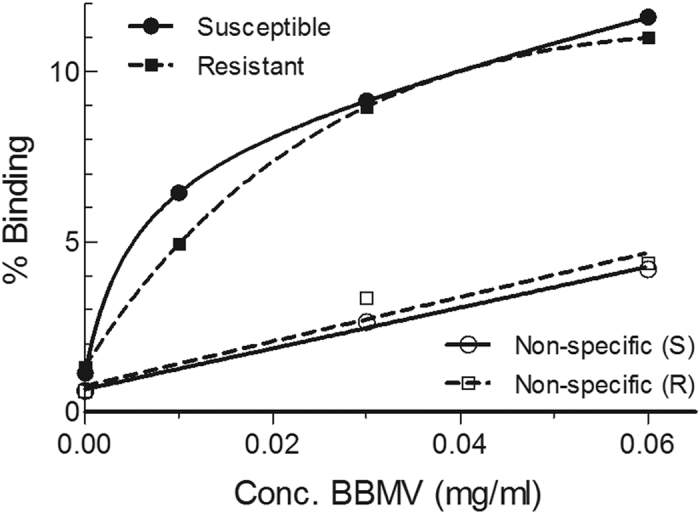
Binding of ^125^I-Vip3Aa to BBMV from GR (susceptible) (●, ○, solid lines) and SP85 (resistant) (◼, ◻, broken lines) colonies at increasing concentrations of BBMV. The figure is representative of two independent experiments with different batches of labeled toxin.

**Figure 3:**
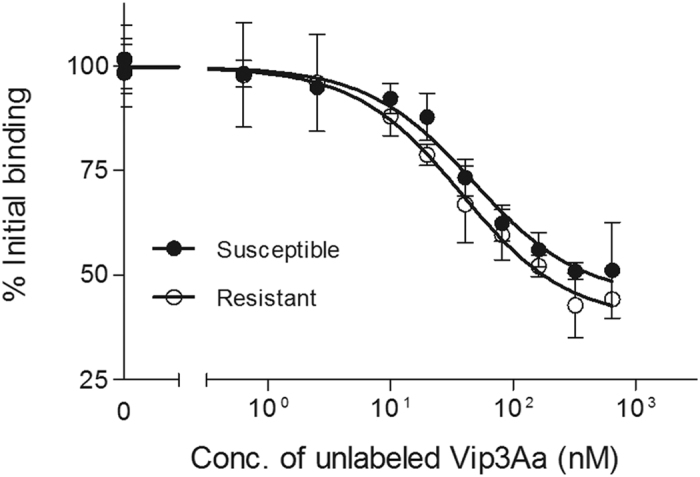
Binding of ^125^I-Vip3Aa to BBMV from resistant and susceptible *H. armigera* at increasing concentrations of unlabeled Vip3Aa. Each data point represents the mean of at least two independent replicates.

Specific binding of ^125^I-Vip3Aa to *H. armigera* BBMV was shown by incubating the labeled toxin in the absence and the presence of an excess of unlabeled Vip3Aa (Fig. 2). The difference between total binding and the binding in the presence of competitor is a measure of the specific binding. BBMV from the two colonies showed specific binding, indicating that the resistance was not due to an absence of binding to the epithelial membrane. To determine whether quantitative binding parameters were significantly different between the two colonies, competition binding assays were performed (Fig. 3). Incubation of a fixed amount of ^125^I-Vip3Aa with increasing concentrations of unlabeled protein showed that Vip3Aa fitted a one-site curve in both the resistant and the susceptible colony. Additionally, there were no significant differences in the equilibrium dissociation constants (*K*_*d*_) and the concentration of binding sites (*R*_*t*_) between the two colonies (Table 3). It is worth mentioning that no specific binding could be obtained with BBMV prepared from lyophilized tissue and, therefore, this type of preparation method does not preserve the binding sites involved in Vip3Aa binding.

## Discussion

In Australia monitoring for resistance to Vip3Aa in field populations of *H. armigera* has been ongoing since 2009 which enabled isolation of resistant alleles and development of colonies with these genes in the laboratory. These colonies will help with the understanding of Vip3Aa resistance in this global pest. This is timely because despite our ability to detect resistance alleles, until recently, *H. armigera* was not exposed to significant selection pressure by Vip3Aa. However, with the recent incursion of *H. armigera* into the New World^2^ there is enormous potential selection for resistance primarily due to the large areas of corn expressing Vip3Aa proteins to control the closely related *Helicoverpa zea* and other lepidopteran corn pests. The bioassays performed herein to characterize Vip3Aa resistance support previous research which demonstrates that resistance alleles can readily be detected in Australian field populations despite no obvious selection (for more detail see Mahon *et al.*^19^). This relatively high baseline level of resistance may reflect selection at a low level from naturally occurring Vip3 toxins and/or direct or indirect (e.g. linkage) selection to something other than Vip3Aa. Our results suggest that natural variation exists in insect populations that could drive resistance once crops expressing Vip3Aa are introduced unless appropriate resistance management measures are implemented (mean expression levels of Vip3Aa in cotton plants is 25 μg/g dry weight, not substantially different from the discriminant dose used in our study)^47^.

The frequency of Vip3Aa resistance alleles in Australian populations of *H. armigera* has not increased significantly in the six seasons that monitoring has taken place, the last four of which are presented herein. F_2_ screens were performed from 2009/10 until 2012/13 and F_1_ screens were performed in 2013/14 and 2014/15. This is not surprising given that products expressing Vip3A have yet to be commercialized. Interestingly, the estimates obtained using F_2_ screens, which can yield false negatives at least for Cry2Ab (S. Downes, unpublished data), are substantially higher than those obtained using F_1_ screens. It is possible that the different *r* frequencies from these methods reflect actual changes in frequencies over time but unlikely given the absence of Bt-crops that could impact on selection for Vip3A resistance (although see above for alternative explanations). Regardless of the reason(s), our data verify that Vip3A resistance alleles exist at relatively high frequencies, and are not rising. This is in contrast to Cry2Ab which has been more variable. In 2010–2012 in particular, Cry2Ab resistance in *H. armigera* doubled compared to the baseline which could have signaled the beginning of a significant resistance problem in response to cotton expressing this toxin^48^ (see also Downes *et al.*^11^ for a similar response in the closely related *H. punctigera*). In the 2014/15 season, the frequency for Cry2Ab declined to baseline levels, which reflects the variability of the presence of this resistance allele, and Cry1Ac resistance remains rare (Downes, unpublished data). For the Vip3Aa alleles detected to date using F_2_ screens, cross-resistance to Cry2Ab and Cry1Ac was assessed and not identified. This is one of the first demonstrations of a lack of cross-resistance to Cry proteins in a Vip3Aa resistant colony, in contrast to previous studies in which Cry-resistant colonies were tested for cross-resistance to Vip3Aa.

A number of the different *H. armigera* Vip3Aa lines were further characterized by performing complementation tests with the first isolated Vip3Aa resistant line (SP85). In the complementation tests, three of them were found to be allelic with the lines previously identified and isolated while the results for another line were less convincing. This suggests that the mechanism present in SP85 is relatively frequent but raises the possibility of other mechanisms/genes being involved in the other F_2_ isolated Vip3A resistant detections. In the case of Cry1Ac and Cry2Ab resistance in *H. armigera*, multiple alleles were identified in the same gene which had the same phenotypic effect^37^,^49^. The fact that the majority of resistant lines tested were allelic allows us to characterize the mechanism of Vip3Aa resistance in a single line and gives us more confidence in extrapolating the findings to the whole population.

The Vip3Aa protein is produced *in planta* as a full length protein of ca. 89 kDa and its purification from cotton leaves indicates that the protein is stored in its protoxin form^47^. Upon ingested by the insect larva, the Vip3Aa protoxin is cleaved by serine proteases to several fragments, with two main products of around 62 kDa and 20 kDa when the incubation is performed under mild conditions (reviewed by Chakroun *et al.*^50^). We chose conditions that yielded the 62 kDa and 20 kDa bands as the main products of the Vip3Aa incubation with *H. armigera* midgut juice to search for differences between the two colonies. Although no differences were observed in the band pattern, the conversion of protoxin (89 kDa) into active toxin (the 62 kDa fragment) was faster, under the same experimental conditions, with midgut juice from the susceptible insects than from their resistant counterparts (Fig. 1). It is difficult to evaluate how this difference in the activation rate may contribute to the resistance to Vip3Aa. In some cases, the kinetics of the protoxin processing to the active toxin has been proposed to be one of the factors determining the potency of Vip3A proteins^22^,^23^,^51^. However, given the narrow spectrum of resistance observed, it is unlikely that a protease-based mechanism is the only factor contributing to the resistance to Vip3Aa^42^,^43^. On the contrary, binding site alteration is a well-documented mechanism of resistance to Cry1A and Cry2A toxins^42^,^43^,^52^. This type of alteration is very specific and cross-resistance is found only in those toxins that bind to the altered binding site.

Specific binding of Vip3A proteins to lepidopteran BBMV has been shown in several insect species using biotin-labeled Vip3Aa competed by unlabeled toxin (reviewed in Chakroun *et al.*^50^), in particular, in *H. armigera*^53^. The use of ^125^I-labeled ligands allows to increase sensitivity and to obtain quantitative results out of the binding assays. Conditions to successfully label Vip3Aa with ^125^I, to perform binding analyses, were set up with *S. frugiperda* BBMV^24^. We have used these conditions and shown specific binding of ^125^I-Vip3Aa to *H. armigera* BBMV (Fig. 2). Equilibrium binding parameters did not show any significant difference between insects from the two colonies (Table 3), indicating that alteration of the binding to the epithelial membrane does not seem to be the reason for the difference in susceptibility of the two insect colonies to Vip3Aa. This result is somewhat unexpected, since binding site alteration confers high levels of resistance to a very small set of structurally related toxins. Several studies have shown that Cry1A and Cry2A toxins do not share binding sites with Vip3A toxins^24^,^25^,^26^,^53^,^54^,^55^. The fact that Vip3Aa-resistant SP85 insects are not cross-resistant to Cry1Ac or Cry2Ab, suggests a highly specific change in the resistant insects but, similarly to other cases of resistance to Cry toxins, this change does not seem to affect binding to the epithelial membrane of the midgut^29^,56,^57^.

In conclusion, alleles for Vip3Aa resistance occur at a relatively high frequency in the field in Australian populations of *H. armigera*, despite the fact that Bt crops expressing this toxin are not yet prevalent in the agroecosystem. Complementation tests with the various alleles isolated by the F_2_ test in isofemale lines indicate that all alleles identified so far are alleles of the same gene. Biochemical analyses of resistant and susceptible insects have shown no differences at the level of binding and minor differences in the activation rate of the Vip3Aa protoxin, which may or may not contribute to resistance. Since the mode of action of Vip3 proteins is not yet well understood, further study with resistant insects may shed light on specific targets of Vip3A proteins which so far are not known.

## Methods

### Insect colonies

The *H. armigera* Vip3Aa resistant colony used in this experiment (SP85) is described in detail elsewhere^19^. Briefly, it is a laboratory colony which was isolated using an F_2_ screen during the summer of 2009–10 from individuals collected as eggs on non-Bt cotton from St. George, Queensland, Australia. This resistant colony was outcrossed five times to the susceptible laboratory colony (GR) to maintain fitness and to produce a colony that was 96.2% isogenic with the susceptible colony. Following each outcross, the colony was reselected with levels of toxin that killed all genotypes except those that were homozygous resistant to Vip3A. All subsequent generations were selected at this dose. The assays reported here were performed with individuals from the 3rd to the 5th generation. However, most of the analyses were conducted with assays on the near-isogenic 5^th^ outcross in order to reduce the potentially misleading effects of hybrid vigor that may be evident when crossing colonies of *H. armigera*.

Individuals from the SP85 colonies survive the maximum concentration of Vip3Aa toxin that can be practically delivered in a surface treatment assay (220 μg/cm^2^) and larvae develop at the same rate as siblings reared on non-treated diet. Resistance for this colony is essentially recessive, with heterozygotes exhibiting concentration-response characteristics that are similar to those of susceptible insects^19^. Reciprocal backcrosses of heterozygotes to resistant colonies produced results for concentration-response assays which confirmed that resistance is essentially recessive –that is, 50% of offspring are homozygous resistant while the remainder are heterozygous and thus phenotypically susceptible^19^. These data are also consistent with the hypothesis that resistance is conferred by a single gene.

The GR colony used in our assays is susceptible to Vip3Aa, Cry1Ac and Cry2Ab toxins. This susceptibility is monitored regularly. The susceptible colony was employed during every screen to verify that a correctly administered discriminating concentration of toxin-containing material was applied. It has been in culture since the mid-1980s and is derived from material collected from cotton fields in the Namoi Valley, northern NSW Australia. On occasions it has been supplemented with additional collections from the same area that were screened for resistance and found to be susceptible.

### Source of toxins

#### Bioassays to characterize Vip3Aa resistance

A Vip3Aa clone in *E. coli* was used as a source of toxin. Production and calibration of the Vip3Aa toxin was described elsewhere^19^.

Cry1Ac toxin was produced by the HD-73 strain of *B. thuringiensis* var. *kurstaki* (producing only the Cry1Ac toxin and spores). Mass production via fermentation of HD-73 was performed by Genesearch (Brisbane, Australia) with a resulting spore/crystal mix. The pellets produced were resuspended and washed three times before use. The extract was used without activating the toxin by trypsin treatment.

Dried and ground corn leaf material was used as a source of Cry2Ab toxin. This corn powder was provided by Monsanto (US) as a lyophilized *Zea mays* leaf powder containing transgenically expressed *B. thuringiensis* crystal protein, Cry2Ab2 at a concentration of 6 mg/g of powder.

#### Biochemical tests

The *E. coli* BL21 expressing Vip3Aa16^58^ used for the biochemical tests was kindly supplied by Dr. Slim Tounsi, CBS (Sfax, Tunisia).

### Bioassays to characterize Vip3Aa resistance

Whole organism bioassays were conducted in 45 well (2.7 cm^2^) trays which contained approximately 2 ml of rearing diet that was overlaid with an aqueous solution of toxin and allowed to air dry. Concentrations were calculated as μg of toxin per cm^2^ of diet surface. After the addition of one neonate larvae per well, trays were heat sealed and maintained at 25 °C and 45–55% RH. Each bioassay consisted of a control (diet with no toxin), plus one toxin concentration. The concentration used was 10 μg of toxin per cm^2^. After 7 days, the larvae were scored as “alive” (exhibiting normal movement) or “dead” (dead, moribund, uncoordinated movement). The mortality of neonates in controls was minimal for all assays (mean mortality 4.1 ± 5%, range 0–11%, *n* = 242 neonate larvae in 6 control assays).

#### Allelism of different isolations of Vip3Aa resistance

Complementation tests were performed after spending 2 to 5 generations (include the two-generation F_2_ tests) in the laboratory. They involved setting up reciprocal crosses between new Vip3Aa-resistant colonies and the SP85 colony. To determine if the characteristics of the captured alleles were similar to those of SP85, the response to a discriminating concentration of toxin in bioassays was determined for the progeny from the above cross and from the parental colonies (SP85, the new resistant colony, and GR). Forty five insects were normally tested in the control per test and the same number exposed to 10 μg/cm^2^ Vip3Aa in 45 well trays (control = 242, tested = 270 in 6 toxin bioassays).

#### Current frequencies of Vip3Aa resistance

Assays to identify resistant insects included F_2_ and F_1_ screens that were conducted using published protocols^12^. We aimed to expose 90 neonate larvae to Vip3Aa toxin for each line.

- (i) F_2_ method. Eggs collected from field hosts of *H. armigera* were reared to pupae. On emergence, single male and female moths were placed in individual 850 ml plastic containers with a dilute honey solution. Eggs laid on the gauze opening of the container were collected every 1–2 days. If they were fertile, around 135 hatchings were reared to establish isofemale lines. On pupation, individuals were sexed and equivalent numbers of males and females were placed in a 5 litre container and allowed to mate.

F_2_ offspring generated from these parents were challenged with a discriminating dose. If either field-collected insect carried a ‘resistant allele’, we would expect at least 6.25% of the toxin-exposed larvae to be homozygous for that allele and thus survive and grow to at least 3^rd^ instar by day 7^59^.

- (ii) F_1_ method. This technique makes use of colonies of resistant insects in a similar fashion to that used by Gould *et al.*^60^ to determine the frequency of resistance in *H. virescens*. Field-collected eggs were reared to pupae and male and female pupae were placed in groups in separate cages. As moths emerged, a male was placed in an 850 ml container with two virgin SP85 females. Similarly, a female was placed in an 850 ml container with two SP85 males.

If fertile eggs were obtained from such crosses, F_1_ offspring were exposed to a discriminating dose. If the field-derived individual tested in this process was heterozygous for resistance, we would expect approximately 50% of the larvae to be homozygous for resistance and therefore to thrive. In the unlikely event that we collected and tested homozygotes from the field, the frequency of survivors would be close to 100%.

#### Cross-resistance to Cry1Ac and Cry2Ab in Vip3A resistant colonies

We present data for a sub-set of isofemale lines that were confirmed to be homozygous for alleles conferring resistance to Vip3A toxin and were challenged in the F_3_ generation as neonates against Cry1Ac and Cry2Ab. The discriminating concentration for Cry1Ac was 0.25 μg/cm^2^ of Cry1Ac delivered in a 50 μl/well solution. After 7 days this concentration killed 95.7 ± 1.8% of a susceptible general rearing colony (*n* = 628 larvae in 10 assays conducted over 7 days) and no surviving larvae grew beyond 2^nd^ instar. The discriminating concentration for Cry2Ab was 1 μg/cm^2^ of Cry2Ab delivered in a 50 μl/ well solution. After 7 days this concentration killed 99.6 ± 0.4% of a susceptible general rearing colony (*n* = 286 larvae in 6 assays conducted over 7 days) and no surviving larvae grew beyond 3^rd^ instar.

### Vip3Aa purification for biochemical analyses

Conditions for bacterial culture and expression of the Vip3Aa16 protein was described previously^22^. For proteolysis assays the expressed Vip3Aa was purified from an *E. coli* cell lysate using a HisTrap FF affinity purification column (GE Healthcare) following the manufacturer instructions. Fractions of 1 ml were eluted from the column and collected in tubes containing 50 μl of 0.1 M EDTA. The most concentrated fractions were pooled and dialyzed against 20 mM Tris, 150 mM NaCl, pH 9, before storage at −20 °C.

For binding assays, Vip3Aa protein was purified as described previously^24^. In brief, the Vip3Aa in the *E. coli* cell lysate was precipitated adjusting the pH to its isoelectric point using acetic acid. The precipitated protein was recovered in the pellet after centrifugation, dissolved in 20 mM Tris-HCl, 150 mM NaCl, pH 9, and treated with 1% trypsin for 2 h at 37 °C. The protein that was used for labeling was further purified by anion-exchange chromatography in an AKTA explorer 100 system (GE Healthcare, UK).

### Midgut juice preparation

Midguts from ten 5^th^ instar larvae of Vip3Aa resistant (SP85) and susceptible (GR) *H. armigera* colonies reared on standard diet were dissected and the peritrophic membrane extracted with its bolus content, which was then homogenized and centrifuged for 10 min at 16000 *g*. The supernatant was collected and distributed in small aliquots, flash frozen in liquid nitrogen and stored in −80 °C. Total protein concentration in the midgut juice was quantified with Bradford reagent using BSA as standard.

### Proteolytic processing of Vip3Aa

Proteolytic processing of Vip3A protoxin by the midgut juice of the susceptible (GR colony) and resistant (SP85 colony) *H. armigera* was first performed with different midgut juice dilutions to select the optimal dilution to perform the kinetic study. To compare the kinetics of Vip3Aa activation by the midgut juice of the susceptible and resistant *H. armigera*, 50 μg of affinity-purified protoxin was incubated with midgut juice at 1/250 (w/w, midgut juice: Vip3Aa) in 70 μl final volume of 20 mM Tris, 150 mM NaCl, pH 9, and incubated for 5, 10, 15, 20, 25, 30 and 60 min at 30 °C. The reaction was stopped by adding the SDS-PAGE loading buffer and heating for 5 min at 99 °C, after which the samples were loaded in 12% polyacrylamide gel. For a quantitative comparison of the processing rate, the amount of Vip3Aa protoxin (89 kDa) and activated toxin (62 kDa) at the different incubation times was quantified densitometrically using the TotalLab 1D *v* 13.01 software. The densitometry values from the 89 kDa and 62 kDa bands were relativized to the input values in each gel, and the background was corrected. Graphical representation was performed using the software GraphPad Prism v 5.00.

### Vip3Aa radiolabeling

Trypsin-activated Vip3Aa was labeled using the chloramine-T method as previously described^61^,^62^. The labeled protein was separated from the excess of iodine by size-exclusion chromatography in a PD10 (GE Healthcare) column. The purity of the labeled protein was checked by analyzing the elution fractions by SDS-PAGE with further exposure of the dried gel to an X-Ray film at −20 °C. The calculated specific activity of the protein was 0.38 mCi/mg.

### BBMV preparation

Fifth-instar larvae of *H. armigera* from both the susceptible (GR) and the resistant (SP85) colony reared on standard diet were dissected and the midguts (without the bolus content) were washed in MET buffer (300 mM mannitol, 5 mM EGTA, 17 mM Tris, pH 7.5) and frozen in liquid nitrogen and preserved at −80 °C until required. Alternatively, midguts in MET buffer were lyophilized and kept at 4 °C^63^. Brush border membrane vesicles (BBMV) were prepared from the frozen or the lyophilized midguts by the differential magnesium precipitation method^63^,^64^, and then frozen in liquid nitrogen, and stored at −80 °C until use. The protein concentration in the BBMV preparations was determined by Bradford^65^ using bovine serum albumin (BSA) as standard.

### Binding assays with ^125^I-labeled Vip3Aa

Prior to use, the buffer of the BBMV was changed to binding buffer (20 mM Tris, 150 mM NaCl, 1 mM MnCl_2_, pH 7.4) supplemented with 0.1% BSA. To determine the appropriate concentration of BBMV to be used for the binding assays, ^125^I-Vip3Aa (1.2 nM) was incubated with increasing amounts of BBMV. An excess of unlabeled Vip3Aa was used to calculate the non-specific binding. The reaction was stopped by centrifuging the tubes at 16,000 *g* for 10 min at 4 °C and the pellet was washed once with 500 μl of cold binding buffer. The radioactivity retained in the pellet was measured in a model 2480 WIZARD^2^ gamma counter.

Competition experiments were performed by incubating 20 μg/ml of BBMV, from both the susceptible and resistant colonies, with 1.2 nM ^125^I-Vip3Aa in 0.1 ml final volume of binding buffer for 90 min at 25 °C in the presence of an increasing amount of unlabeled Vip3Aa protein. The reaction was stopped and the remaining radioactivity measured as described above. The dissociation constant (*K*_*d*_) and the concentration of binding sites (*R*_*t*_) were calculated using the LIGAND program^66^.

## Additional Information

**How to cite this article**: Chakroun, M. *et al.* Characterization of the resistance to Vip3Aa in *Helicoverpa armigera* from Australia and the role of midgut processing and receptor binding. *Sci. Rep.* **6**, 24311; doi: 10.1038/srep24311 (2016).

## Figures and Tables

**Table 1:** Complementation testing of four field isolated Vip3Aa resistant *H armigera* colonies with the SP85 type colony.

Year isolated	Colony crossed to SP85	Exposure	Dead	Alive	Total	% Dead
2012	11–1112	buffer	0	20	20	0.0
		Vip3Aa	5	20	25	20.0
2012	11–2201	buffer	0	45	45	0.0
		Vip3Aa	27	18	45	60.0
2013	12–2602	buffer	5	40	45	11.1
		Vip3Aa	7	38	45	15.6
2013	12–2998	buffer	0	45	45	0.0
		Vip3Aa	0	45	45	0.0
Various	Resistant (SP85)	buffer	0	45	45	0.0
		Vip3Aa	0	45	45	0.0
Various	Susceptible (GR)	buffer	0	45	45	0.0
		Vip3Aa	45	0	45	100.0

All crosses were tested with the discriminating dose of Vip3Aa (10 μg/cm^2^) and scored for survival.

**Table 2:** A sample of isofemale lines generated from F_2_ screens that were confirmed to be homozygous resistant for Vip3Aa resistance, and their responses to Cry1Ac and Cry2Ab toxin in the F_3_ generation.

Isofemaleline	Cry1Ac			Cry2Ab		
	Dead	Tested	% 3rd	Dead	Tested	% 3rd
9.1809	90	90	0.00	85	88	0.00
9.2542	90	90	0.00	85	90	0.00
10.1634	90	90	0.00	90	90	0.00
10.1743	90	90	0.00	90	90	0.00
10.173	90	90	0.00	89	89	0.00
11.1013	82	86	0.00	82	88	0.00
11.1112	89	90	0.00	0	90	0.00
11.2731	90	90	0.00	90	90	0.00
11.3132	87	90	0.00	90	90	0.00
12.2169	90	90	0.00	78	88	0.00
12.2602	89	89	0.00	88	89	0.00
12.2696	90	90	0.00	90	90	0.00
12.3256	90	90	0.00	90	90	0.00

Assays were performed on neonates. After 7 days they were scored as being alive and at least 3^rd^ instar, or dead or not at 3^rd^ instar.

**Table 3:** Equilibrium dissociation constant (*K*_*d*_) and concentration of binding sites (*R*_*t*_) of Vip3Aa with BBMV from susceptible and resistant *H. armigera.*

Protein	Mean ± SEM	*R*_*t*_ (pmol/mg)
	*K*_*d*_ (nM)	
Susceptible	25 ± 4	139 ± 33
Resistant	19 ± 5	141 ± 25
